# Neoadjuvant Immunotherapy in Resectable Non-Small Cell Lung Cancer. A Narrative Review

**DOI:** 10.3390/life11101036

**Published:** 2021-10-01

**Authors:** Lavinia Gatteschi, Mauro Iannopollo, Alessandro Gonfiotti

**Affiliations:** 1 Thoracic Surgery Unit, Cardiothoracic Department, University of Florence, 50100 Florence, Italy; a.gonfiotti@unifi.it; 2 Oncology Unit, San Jacopo Hospital, Oncology Department, Central Tuscany AUSL, 51100 Pistoia, Italy; mauro.iannopollo@uslcentro.toscana.it

**Keywords:** resectable NSCLC, neoadjuvant immunotherapy, immune checkpoint inhibitors

## Abstract

Lung cancer is one of the most common malignant tumors and it is the leading cause of cancer-related mortality worldwide. For early-stage Non-Small Cell Lung Cancer (NSCLC), surgical resection is the treatment of choice, but the 5-year survival is still unsatisfying, ranging from 60% to 36% depending on the disease stage. Multimodality treatment with adjuvant chemotherapy did not lead to clinically relevant results, improving survival rates by only 5%. Recently, immune checkpoint inhibitors (ICIs) are being studied as neoadjuvant treatment for resectable NSCLC too, after the satisfactory results obtained in stage IV disease. Several clinical trials are evaluating the safety and feasibility of neoadjuvant immunotherapy and their early findings suggest that ICIs could be better tolerated than standard neoadjuvant chemotherapy and more effective in reducing cancer local recurrence and metastasis. The aim of this review is to retrace the most relevant results of the completed and the ongoing clinical trials, in terms of efficacy and safety, but also to face the open challenges regarding ICIs in neoadjuvant setting for resectable NSCLC.

## 1. Introduction

### 1.1. Background

Lung cancer is one of the most common malignant tumors and it is the leading cause of cancer-related mortality worldwide, with 1.76 million victims annually [1]. Non-Small Cell Lung Cancer (NSCLC) accounts for about 80–85% of them [2]. Thanks to the spread of computed tomography (CT) and international screening programs, an increasing number of patients receive their diagnosis in the early stage, when surgical resection with curative intent is still possible and represents the best chance of cure [3].

However, the 5-year survival rates of these patients are still unsatisfying, ranging from 60% in stage IIA to 36% in stage IIIA [4]. Moreover, between 30% and 60% of them will develop a metastatic disease after radical resection [5].

Moreover, it is now common knowledge that resectable NSCLC benefits from a multimodality treatment rather than surgery alone. Adjuvant chemotherapy, for example, has been shown to improve 5-year survival rates by around 5%, though it cannot be defined as a successfully achieved goal [6].

Moreover, the neoadjuvant trials showed that neoadjuvant standard chemotherapy followed by surgery for stage I to III NSCLC improved the 5-year OS by 5% (40–45%) (HR = 0.87, 95% CI: 0.78–0.96, *p* = 0.007) compared to surgery alone [6].

At the same time, we have limited evidence regarding the efficacy of the induction chemoradiotherapy (CRT) followed by surgery. The INT0139 study, a phase 3 trial, compared the standard of CRT vs. induction CRT (45 Gy) followed by surgery for pathologically diagnosed cN2 resectable NSCLC [7]. In an exploratory subset analysis, pneumonectomy after CRT induction was associated with a treatment-related mortality rate of 26% and a worse OS than radical CRT. However, lobectomy after CRT induction was associated with a treatment-related mortality rate of 1% and significantly improved OS compared to radical CRT (median OS, 33.6 vs. 21.7%, *p* = 0.002).

ICIs improve the prognosis of patients with stage IV NSCLC [8,9,10,11,12,13]. These results have encouraged the anticipated use of immunotherapy in a setting of adjuvant, neoadjuvant therapy. Several large-scale phase 3 studies are in progress in an adjuvant setting, investigating the efficacy of ICIs after complete resection in patients with pathological stage IB to IIIA NSCLC. There are currently studies including ICI monotherapies and combination therapies of ICI and conventional chemotherapy.

Recently, the IMpower010 trial revealed that adjuvant chemotherapy followed by maintenance with atezolizumab showed the significant prolongation of disease-free survival (DFS) in patients with PD-L1 TC ≥ 1% stage II to IIIA (HR, 0.66; 95% CI, 0.50–0.88) NSCLC [14].

Neoadjuvant therapy may control micrometastases in the early phases and may offer an opportunity to evaluate drug sensitivity. Adjuvant therapy may or may not be performed if the patients are not fit for chemotherapy after surgery. Neoadjuvant therapy can be performed with good compliance, but may cause increased postoperative complications and treatment-related adverse events.

This is why there is an urgent and unmet need to seek novel and more effective treatments for NSCLC, such as neoadjuvant therapy. In particular, neoadjuvant immunotherapy with immune checkpoint inhibitors (ICIs) is being explored in an increasing number of studies and clinical trials that are moving from metastatic disease to early-stage NSCLC looking for efficacy in resectable patients too.

### 1.2. The Rationale of Neoadjuvant Immunotherapy in NSCLC

ICIs have been successfully used against many solid tumors such as triple-negative breast cancer, melanoma, and urothelial carcinoma [15,16,17]. We can say that the PACIFIC trial definitely demonstrated the efficacy and the great potential of immunotherapy in lung cancer [18]. In this prospective randomized trial patients with unresectable stage III lung cancer were randomized after chemo-radiation therapy to durvalumab or placebo. After 24 months of follow-up, the overall survival rate was significantly higher in patients who received durvalumab versus the ones matched to placebo, 66.3% and 55.6%, respectively. Moreover, the trial highlighted that 85% of patients treated with durvalumab who underwent disease progression presented local recurrence (lung or regional lymph nodes).

The clinical data from this trial stated not only the efficacy of durvalumab in prolonging the overall survival of non-resectable NSCLC, but also suggested that immunotherapy could be applied in a multimodality treatment alongside surgery too, thanks to its control over distant metastases.

The rationale of using immunotherapy against NSCLC (and any other type of malignancies) lies in the clinical need to inhibit the tumoral pathways that downregulate the patients’ immune T-cell response. The majority of the drugs used (durvalumab, pembrolizumab, nivolumab, and atezolizumab) modulate the interaction of programmed cell death protein 1 (PD-1)/programmed cell death protein 1 ligand (PD-L1). The antibodies lead to the blockade of this immunosuppressive interaction and allow the patient’s T cells to recognize the antigen-presenting tumor cells [19].

Ipilimumab, on the other side, modulates the cytotoxic T-lymphocyte-associated protein 4 (CTLA-4) to block its pathway and to ensure the immune T-cell response [20].

This is why the use of immunotherapy in the neoadjuvant setting seems a reasonable and effective application, in fact, the entire tumor exhibits a high number of antigens that can be linked to the antigen-presenting cells to induce a stronger and prolonged immune T-cell response, preventing tumor recurrence.

The majority of the advantages in administering ICIs as neoadjuvant therapy have been highlighted by Liu and colleagues in their preclinical study [21]. The authors used two immunocompetent murine models of triple-negative breast cancer and treated them with neoadjuvant or adjuvant immunotherapy (anti-PD-1 alone or in combination with anti-CD137). The study showed that the group treated with neoadjuvant immunotherapy had a long-term survival of 40% versus 0% of the adjuvant group. Moreover, the peripheral blood examination of the neoadjuvant group displayed a significantly higher number of tumor-specific CD8+ T cells compared to the adjuvant group.

There are several more benefits that make neoadjuvant immunotherapy a suitable treatment option.

First of all, one of the goals of administering neoadjuvant ICIs is to reduce the size of the primary tumor and, as a result, to increase the chance of radical surgical resection, but also to control and ideally eradicate the circulant micrometastases. In fact, activated T cells run through the lymphatic and the bloodstream both to the primary tumor and against micrometastatic sites to perform their tumor-killing effect [22]. The rationale of administering ICIs before surgery also lies in the integrity of the blood and lymphatic flow that can lead the activated cells to the tumor site unhindered.

Moreover, the interaction of these drugs with the microenvironment of the tumor induces, among other things, a devascularization of the tumor itself, that may result in adhesions and fibrotic retraction. This effect may represent the downside of neoadjuvant immunotherapy, leading to a more challenging surgical field, as well as after neoadjuvant chemotherapy.

In addition, administering drugs to a patient with an intact immune system gives earlier endpoints to assess the patient’s response to therapy in terms of sensitivity or resistance to it. The data collected during this phase of the therapeutic pathway enable a more accurate choice of the most appropriate treatment regimen after surgery and ineffective agents can be stopped and substituted with alternatives.

Moreover, even if immunotherapy showed some adverse effects such as pneumonia, myocarditis, and neuromuscular toxicity [23,24] their impact seems significantly lower and better tolerated compared to the toxicity related to traditional chemo-radiation therapy.

Neoadjuvant immunotherapy may also shorten clinical trials and lead to a more diffuse and widely accepted use of surrogate predictors of overall survival, such as major pathological response (MPR) and pathological complete response (pCR).

These short-term efficacy indicators are increasingly used as primary endpoints in the majority of completed and ongoing clinical trials.

MPR, for example, has been found to correlate with long-term survival in patients with NSCLC treated with neoadjuvant chemotherapy, and its use is accepted as a reliable endpoint in this setting [25,26].

The use of these surrogates may fasten the approval of immunotherapy agents too and they are defined as follows:

MPR: The resected specimen presents ≤ 10% of viable tumor cells. It is the most commonly used in the ongoing trials;
pCR: The evaluation of the specimen and the regional lymph nodes does not detect any residual invasive cancer [25]. Unfortunately, pCR is rarely reached in NSCLC; thus it is not such a feasible endpoint to assess the efficacy of neoadjuvant immunotherapy.

The aim of this review is to examine the clinical results of the most relevant studies on neoadjuvant immunotherapy in resectable NSCLC and to discuss the emerging data from the most innovative ongoing clinical trials. We will also focus on the potential problems related to this new approach.

## 2. Materials and Methods

Clinical data and results were found by searching PubMed for articles only in the English language from 2018 to 2021. The keywords searched were neoadjuvant immunotherapy, early-stage NSCLC, resectable lung cancer. Moreover, clinicaltrials.gov was searched by inserting the words neoadjuvant immunotherapy in NSCLC. In this review, we decided to cite the more relevant studies after an accurate screening of the ones found.

## 3. Results

### 3.1. The Results of Clinical Trials

The results of the most relevant clinical trials using neoadjuvant immunotherapy alone are depicted in Table 1.

The CheckMate 159 by Forde and colleagues [27] explored for the first time in a prospective trial the efficacy of neoadjuvant immunotherapy for NSCLC. The study enrolled 22 patients with resectable (stage IB-IIIA) NSCLC to receive two cycles of nivolumab before surgery. The results were promising: 9 patients out of the 20 (45%) who received the planned therapy achieved MPR and none of them presented delay in surgery.

The Lung Cancer Mutation Consortium conducted the LCMC3 trial [28] where two cycles of atezolizumab were administered to 181 patients with stage IB-IIIA and selected IIIB. The primary endpoint was MPR which was received by 30 patients (21%). Grade 3 or greater treatment-related adverse events manifested in 6% of patients and 22 (12%) did not undergo surgery. It is interesting to underline that the MPR of patients expressing more than 50% of PD-L1 was 33% versus 11% of patients with expression lower than 50%.

The IONESCO trial [29] set as the primary endpoint the complete surgical resection (R0 according to RECIST 1.1 criteria) in patients with stage IB-IIIA NSCLC after neoadjuvant durvalumab. The study was stopped before the scheduled date because of a high 90-day postoperative (4 patients, 9%). Actually, none of these deaths was related to immunotherapy treatment.

Neoadjuvant immunotherapy has been and is being tested synergically with chemotherapy. Clinical trials evaluating neoadjuvant immunotherapy in combination with chemotherapy and with or without adjuvant therapy are reported in Table 2.

The NADIM trial by Provencio and colleagues [30] assessed the safety and feasibility of neoadjuvant nivolumab plus carboplatin and paclitaxel in 46 patients with stage IIIA NSCLC. After surgery, one year of adjuvant nivolumab was administered too. A total of 89% of the study population underwent surgery and MPR was achieved in 83% of patients. After 18 months of follow-up, the progression-free survival rate was 87%. A total of 93% of patients presented treatment-related adverse events during neoadjuvant therapy, such as nausea, alopecia, fatigue, and neurotoxicity, but these did not lead to treatment interruption or delay in surgery.

Neoadjuvant immunotherapy is being tested not only in monotherapy or in combination with standard chemotherapy, but dual immunotherapy is being investigated too. The phase 2 randomized trial NEOSTAR [31], for example, used neoadjuvant nivolumab or a combination of nivolumab and ipilimumab in 44 patients with resectable NSCLC, stage I-IIIA. The primary endpoint was MPR and it was evaluated individually in the two arms of the study. Out of the 37 patients who underwent surgery, 8 patients of the 16 (50%) treated with nivolumab and ipilimumab achieved MPR, while the nivolumab arm presented a 24% (5/21) MPR rate. Compared with nivolumab, the association between nivolumab and ipilimumab was shown to be superior in terms of higher pCR too (10% versus 38%) and it seems to enhance immunologic response and memory. This data suggested that combination immunotherapy could be more effective than single drug immunotherapy, but could be related to higher toxicity.

### 3.2. The Ongoing Clinical Trials

The majority of clinical trials investigating neoadjuvant immunotherapy are still ongoing and only partial results are being released. The current studies are focusing not only on single drug neoadjuvant immunotherapy, but also on neoadjuvant chemo-immunotherapy and on multimodality treatment with neoadjuvant and following surgery adjuvant immunotherapy. The endpoints are still heterogeneous and evaluate the efficacy of neoadjuvant immunotherapy through survival surrogates (MPR, pCR) and the safety and feasibility of ICIs.

The PRINCEPS trial enrolled 30 patients with clinical stage IA-IIIA resectable NSCLC [32]. The primary endpoint was the rate of toxicities and morbidities after one month of surgery in patients who received one cycle of neoadjuvant atezolizumab. None of them were delayed in surgery and 29 received complete resection (R0). In contrast with other trials, no MPR was observed, but this could be explained by the short delay between the infusion of atezolizumab and surgery, which happened from 21 to 28 days after.

Moreover, the trial proved again the safety and feasibility of neoadjuvant immunotherapy, in fact, there was only one treatment-related adverse effect, a grade 1 parietal pain.

The NEOMUN trial is a single-arm monocentric study that aims to study the safety and feasibility of neoadjuvant pembrolizumab in patients with resectable NSCLC stage II-IIIA [33]. The first clinical results enrolled 15 patients who, after completion of immunotherapy, underwent surgery with curative intent. In this phase, 13 patients received the scheduled immunotherapy and 4 (27%) of them reached MPR. Moreover, the study investigated the clinical response too by the decreasing PET activity of the tumor, which was detected in the same four patients.

Grade 2–3 treatment-related adverse events happened in five patients (33%), the overall postoperative morbidity was 7% and 30-day mortality was 0%. In conclusion, neoadjuvant pembrolizumab resulted as a feasible and safe treatment.

Shu and colleagues assessed four cycles of atezolizumab plus carboplatin and nab-paclitaxel in a neoadjuvant setting in 30 patients with stage IB-IIIA NSCLC [34]. The primary endpoint was MPR and at the cutoff data and it was achieved by 17 patients (57%). In this phase II trial, the most common treatment-related adverse events (grades 3–4) were neutropenia and thrombocytopenia.

CheckMate 816 (NCT02998528) is a phase 3 randomized multicenter trial whose results have been recently announced. It randomized 358 patients with resectable NSCLC to receive three cycles of neoadjuvant nivolumab and histology-based platinum doublet chemotherapy or neoadjuvant chemotherapy alone. The primary endpoints are pCR and event-free survival (EFS). The group treated with neoadjuvant nivolumab reached 24% of pCR versus only 2.2% of the group matched to platinum doublet alone. The significant improvement in pCR is the first to be demonstrated in patients with NSCLC treated in a multimodality treatment with neoadjuvant immunotherapy and it is not related to PD-L1 expression. One of the secondary endpoints is MPR, which is reached in 36.9% of patients treated with nivolumab, while patients undergoing chemotherapy alone reached an 8.9% MPR rate.

Moreover, 83% of patients who received nivolumab underwent surgery and achieved a complete surgical resection (R0). Surgery-related and treatment-related adverse events were similar in both arms of the study. In conclusion, CheckMate 816 demonstrated that neoadjuvant chemo-immunotherapy does not affect the feasibility of surgery and meanwhile increases pCR.

Another phase 3 trial is the Aegean trial (NCT03800134), which is a double-blind multicenter study. It is still recruiting patients and it aims to randomize approximately 800 patients with stage II and III NSCLC to receive neoadjuvant durvalumab or placebo and concurrent platinum-based chemotherapy and adjuvant chemotherapy with durvalumab or placebo.

The primary endpoint is pCR, while secondary endpoints comprehend safety assessments, MPR, and overall survival. The estimated study completion date is April 2024.

It is also worth citing the NeoCOAST trial (NCT03794544), which evaluates in patients with stage I-IIIA NSCLC the safety of neoadjuvant durvalumab alone or in combination with novel agents oleclumab (+MEDI9447), monalizumab (IPH2201), and danvatirsen (AZD9150). The primary endpoint is MPR and the secondary includes feasibility of tumor resection and pCR. The study ended in January 2021 and its results will be likely published soon.

## 4. Discussion

An increasing number of trials are demonstrating the feasibility and safety of immunotherapy in neoadjuvant settings against NSCLC. Furthermore, many trials are still recruiting and their first results are encouraging.

On the other hand, the majority of the collected data use surrogate predictors of survival, while the goal is to assess the impact of neoadjuvant immunotherapy in terms of overall survival of patients. This lack of uniformity in clinical trials endpoints could lead to heterogeneous results that are difficult to compare. On the other side, there is an unmet need to discover new and more effective treatments for NSCLC, which is the leading cause of cancer-related mortality worldwide and it could be counterproductive to assess long-term endpoints of five years or more. This is why pCR and, more frequently, MPR are currently used to predict patients’ response to neoadjuvant therapy, but cannot provide long-term disease monitoring which will be necessary to definitely validate the efficacy of neoadjuvant ICIs in NSCLC.

Despite the encouraging results from the clinical trials, there are several challenges still to solve.

For example, PD-L1 expression level, which is the main biomarker of ICIs, has to be further investigated, since studies reported conflicting results on its role in predicting the curative effect of the drugs mentioned above. In fact, while in 2014 Velcheti and colleagues [35] stated that higher PD-L1 expression led to higher effectiveness of ICIs, LCMC3, and NEOSTAR showed treatment response in both PD-L1 positive and negative NSCLC. MPR in LCMC3 was significantly higher in patients with PD-L1 expression, >50% than in patients with lower protein expression.

Timing and the model of neoadjuvant immunotherapy are still a matter of debate too. Clinical trials are simultaneously evaluating the efficacy of single drug neoadjuvant immunotherapy, a combination of different neoadjuvant ICIs and ICIs with standard chemotherapy, as well as adjuvant chemotherapy and maintenance immunotherapy.

In conclusion, the data collected show that ICIs induce promising degrees of MPR (up to 45% alone and even up to 83% combined with chemotherapy) indicating that induction immunotherapy is now emerging as the new effective standard of care for patients with resectable NSCLC. The definition of the better schedule and patient selection remain fundamental to optimize the therapeutic effectiveness and, in addition, to limit useless therapy.

## Figures and Tables

**Table 1:** Clinical trials with neoadjuvant immunotherapy alone.

Trial	NSCLC Stage	Drug	Primary Endpoint	MPR (%)
NCT02259621 (Checkmate 159)	IB-IIIA	Nivolumab	Safety, Feasibility	45%
NCT02927301 (LCMC3)	IB-IIIB	Atezolizumab	MPR	33% for PD-L1 > 50%
NCT03030131 (IONESCO)	IB-IIIA	Durvalumab	% of R0	19%
NCT03158129 (NEOSTAR)	I-IIIA	Nivolumab +/− Ipilimumab	MPR	50% vs. 24%
NCT02994576 (PRINCEPS)	I-IIIA	Atezolizumab	Toxicity	14%
NCT03197467 (NEOMUN)	II-IIIA	Pembrolizumab	Safety	27%

**Table 2:** Clinical trials with neoadjuvant immunotherapy in combination with chemotherapy and with or without adjuvant therapy. Abbreviations: NP—not provided.

Trial	NSCLC Stage	Drug	Primary Endpoint	MPR (%)	Adjuvant Therapy
NCT03081689 (NADIM)	IIIA	Nivolumab + chemotherapy	Safety, Feasibility	83%	Nivolumab
NCT02716038 (Shu et al.)	IB-IIIA	Atezolizumab + chemotherapy	MPR	57%	NP
NCT02998528 (CheckMate 816)	IB-IIIA	Nivolumab + chemotherapy	EFS, pCR	36.9%	NP
NCT03800134 (AEGEAN)	II-IIIA	Durvalumab + chemotherapy	MPR, EFS	Unpublished	Chemotherapy +/− durvalumab
NCT03794544 (NeoCOAST)	I-IIIA	Durvalumab + Oleclumab/Monalizumab/Danvatirsen	MPR	Unpublished	NP

## Data Availability

Data sharing is not applicable to this article.
